# Crucial roles of Robo proteins in midline crossing of cerebellofugal axons and lack of their up-regulation after midline crossing

**DOI:** 10.1186/1749-8104-3-29

**Published:** 2008-11-05

**Authors:** Atsushi Tamada, Tatsuro Kumada, Yan Zhu, Tomoko Matsumoto, Yumiko Hatanaka, Keiko Muguruma, Zhe Chen, Yasuto Tanabe, Makio Torigoe, Kenta Yamauchi, Hiroshi Oyama, Kazuhiko Nishida, Fujio Murakami

**Affiliations:** 1 National Institute for Basic Biology, Myodaiji-cho, Okazaki 444-8585, Japan; 2 RIKEN Brain Science Institute, 2-1 Hirosawa, Wako 351-0198, Japan; 3 CREST, JST (Japan Science and Technology), Kawauguchi, 332-0012, Japan; 4 Hamamatsu University School of Medicine, 1-20-1, Handayama, Hamamatsu, Shizuoka, 431-3192, Japan; 5 SORST, JST, Kawauguchi, 332-0012, Japan; 6 Graduate School of Frontier Biosciences, Osaka University, Suita, Osaka 560-8531, Japan; 7 Nara Institute of Science and Technology, 8916-5, Takayama-cho, Ikoma, Nara 630-0192, Japan; 8 RIKEN Center for Developmental Biology, 2-2-3 Minatojima-Minamimachi, Chuo, Kobe 650-0047, Japan; 9 Division of Research, Genentech Inc, South San Francisco, CA 94080, USA

## Abstract

**Background:**

Robo1, Robo2 and Rig-1 (Robo3), members of the Robo protein family, are candidate receptors for the chemorepellents Slit and are known to play a crucial role in commissural axon guidance in the spinal cord. However, their roles at other axial levels remain unknown. Here we examine expression of Robo proteins by cerebellofugal (CF) commissural axons in the rostral hindbrain and investigate their roles in CF axon pathfinding by analysing Robo knockout mice.

**Results:**

We analysed the expression of Robo proteins by CF axons originating from deep cerebellar neurons in rodent embryos, focusing on developmental stages of their midline crossing and post-crossing navigation. At the stage of CF axon midline crossing, mRNAs of Robo1 and Robo2 are expressed in the nuclear transitory zone of the cerebellum, where the primordium of the deep cerebellar nuclei are located, supporting the notion that CF axons express Robo1 and Robo2. Indeed, immunohistochemical analysis of CF axons labelled by electroporation to deep cerebellar nuclei neurons indicates that Robo1 protein, and possibly also Robo2 protein, is expressed by CF axons crossing the midline. However, weak or no expression of these proteins is found on the longitudinal portion of CF axons. In *Robo1*/*2 *double knockout mice, many CF axons reach the midline but fail to exit it. We find that CF axons express Rig-1 (Robo3) before they reach the midline but not after the longitudinal turn. Consistent with this *in vivo *observation, axons elicited from a cerebellar explant in co-culture with a floor plate explant express Rig-1. In *Rig-1 *deficient mouse embryos, CF axons appear to project ipsilaterally without reaching the midline.

**Conclusion:**

These results indicate that Robo1, Robo2 or both are required for midline exit of CF axons. In contrast, Rig-1 is required for their approach to the midline. However, post-crossing up-regulation of these proteins, which plays an important role in spinal commissural axon guidance, does not appear to be required for the longitudinal navigation of CF axons after midline crossing. Our results illustrate that although common mechanisms operate for midline crossing at different axial levels, significant variation exists in post-crossing navigation.

## Background

In the bilaterally symmetrical central nervous system, information transfer between both sides of the body is mediated by commissural neurons. Commissural axons that cross the ventral midline of the hindbrain and the spinal cord show a stereotyped growth behavior during development: They initially grow straight toward the midline along the circumferential axis, but after midline crossing turn at a right angle to grow along the longitudinal axis (reviewed in [1,2]).

The floor plate (FP) at the ventral midline plays a key role in guiding commissural axons between the spinal cord and the hindbrain. At both axial levels, the FP located at the ventral midline of the neural tube attracts commissural axons by releasing the chemoattractant Netrin-1 [3-7]. After arriving at the FP, commissural axons continue growing across the FP without stalling. This is because commissural axons change their responsiveness to the FP chemoattractant [8] and chemorepellents [9,10].

In the *Drosophila *ventral nerve cord, Robo, a receptor for chemorepellent Slits concentrated around the midline, controls commissural axon midline crossing. Commissural axons express low levels of Robo before they cross the midline but up-regulate Robo after crossing [11], augmenting a repulsive response to Slits [12]. Consistent with this idea, post-crossing but not pre-crossing axons of a rodent spinal cord explant are inhibited by Slit2 [10]. Genetic analysis supports the involvement of the Slit/Robo system in spinal cord commissural axon guidance. In *Slit1*, *Slit2 *and *Slit3 *triple knockout mice as well as in *Robo1 *single mutant mice, fewer commissural axons exit the midline [13]. Robo1 and Robo2 are expressed at low levels by pre-crossing commissural axons but are highly up-regulated after crossing [14]. Moreover, removal of Rig-1 (Robo3), which is expressed by a pre-crossing portion of commissural axons, prevents midline crossing of commissural axons [14,15] suggesting that Rig-1 plays a pivotal role in axonal guidance in the spinal cord. Genetic and *in vitro *analysis showed that Rig-1 functions to repress axon sensitivity to Slits [14].

Despite the abundance of evidence describing the important role of Robo proteins in midline crossing of spinal commissural axons, their role at other axial levels remains unknown. Therefore, the aim of the present study was to uncover whether a common molecular mechanism guides commissural axons at other axial levels. To this end, we examined the expression patterns of Robo proteins in cerebellofugal (CF) axons, rostral hindbrain commissural axons originating from the deep cerebellar nuclei, and the guidance of CF axons in *Rig-1 *knockout mice and *Robo1*/*2 *double knockout mice. We find that in Rig-1 deficient mouse embryos, CF axons extend longitudinally on the ipsilateral side, failing to cross the midline. In *Robo1*/*2 *double knockout mice, many CF axons reach the midline but fail to exit it. Curiously, expression of Robo proteins does not seem to be up-regulated in post-crossing CF axons, unlike the spinal cord commissural axons. These results suggest that Robo proteins are crucial regulators of midline crossing but may not be essential for post-crossing longitudinal navigation in developing CF axons.

## Results

In the rat, CF axons initiate growth at embryonic day (E)12–13, reach the ventral midline at E14, and then execute rostral and caudal turns at E15–16 [5].

### Generation of specific antibodies against Robo1, Robo2 and Rig-1 proteins

To determine the precise localization of Robo1, Robo2 and Rig-1 proteins on CF axons before and after midline crossing, we generated antibodies against these three Robo proteins. The antibodies described here were first reported in Sabatier *et al*. [14] and have been successfully used in several other studies [13,14,16-19], although detailed methods of antibody generation and characterization of their specificity have not been reported. We prepared recombinant Fc fusion proteins of the ectodomains of rat Robo1 (Robo1eFc), rat Robo2 (Robo2eFc) and mouse Rig-1 (Rig-1eFc), and immunized rabbits with them. Immunoblot analysis showed that each Robo antibody recognized a major band at about 160 kDa of the corresponding recombinant Robo-Fc protein, which was also recognized by the anti-Fc antibody (Additional files 1 and 2). Specificity of the antibodies was confirmed by immunohistochemistry with antibodies pre-absorbed with Robo2eFc, Rig-1eFc, or Fc protein and observations of immunoreactivities in *Rig-1 *or *Robo1*/*2 *double knockout mice preparations.

### Expression patterns of Robo1 and Robo2 proteins in the rostral hindbrain

To examine the expression patterns of Robo1 and Robo2 proteins in the region corresponding to the CF axonal tracts, immunohistochemical analyses with anti-Robo1 and anti-Robo2 antibodies was performed using flat, whole-mount preparations of the rat rostral hindbrain from E13 to E16. In the descriptions hereafter, the terms 'circumferential axis' and 'longitudinal axis' refer to the axis along the dorsoventral and rostrocaudal axes, respectively.

**Figure 1:**
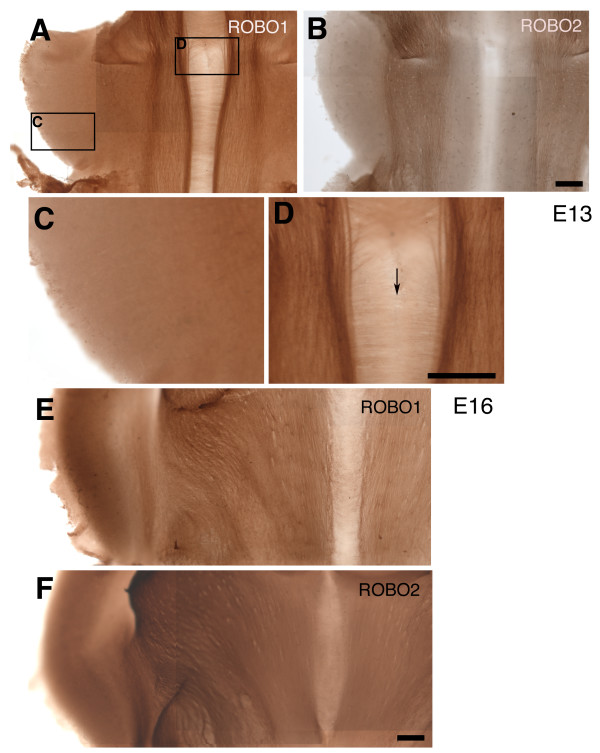
**Expression patterns of Robo1 and Robo2 proteins in the rostral hindbrain**. **(A-F) **Embryonic rat hindbrain was flat, whole-mounted and immunostained for Robo1 (A,C,D,E) and Robo2 (B,F). The whole-mount preparations were placed with the ventricular side down. (A-D) are from embryonic day (E)13 and (E-F) are from E16 rat embryos. Both Robo1 and Robo2 proteins are largely expressed by longitudinally growing axons. (A) At E13, when cerebellofugal axons have just left the cerebellar plate (CP) [5], strong expression of Robo1 is seen in longitudinal axons. (C,D) Higher magnifications of areas shown by rectangles in (A) in the CP (C) and the midline floor plate (FP) (D), respectively. A small number of midline-crossing axons can be seen in the FP (arrow in D). (B) Immunostaining for Robo2. Robo2 immunopositive fibres also run longitudinally but in a region far from the FP. At E16, both Robo1 and Robo2 immunopositive fibres run more or less longitudinally in overlapping regions (E,F). Scale bars = 200 μm; the bar in (B) also applies to (A); that in (D) also applies to (C); and that in (F) also applies to (E).

In most cases, Robo1 and Robo2 immunoreactivities were observed on the axonal processes but not in the cell soma. At around E13, the CF axons have just left the cerebellar plate (CP), and started to grow circumferentially toward the FP [5]. At this stage, Robo1 immunoreactivity was largely confined to longitudinally extending axonal profiles (Figure 1A). Immunoreactivity was not found in the CP where the cell bodies of prospective deep cerebellar neurons should be located, suggesting minimal Robo1 expression on the cell body (Figure 1C). Near the FP, Robo1 immunoreactive fibres were densely distributed, running longitudinally (Figure 1D), while only a weak immunoreactivity was found on some circumferentially growing axons (Figure 1D, arrow). The latter are unlikely to be CF axons because these axons had not arrived at the midline at this stage [5].

Like Robo1, Robo2 immunoreactivity was also found in the rostral hindbrain (Figure 1B) but not in the CP. At E13, most Robo2 immunopositive fibres also extended longitudinally, but were excluded from the ventral (medial) neural tube. Furthermore, they ran more dorsally (laterally) compared to Robo1 immunoreactive fibres. As development proceeded, both Robo1 and Robo2 immunoreactivities were detected in a wider area in the rostral hindbrain, but were still confined mainly to longitudinally growing fibres (data not shown). At E16, when the CF axons execute a longitudinal turn in the medial region [5], both Robo1 and Robo2 immunopositive fibres were more marked in the lateral half of the rostral hindbrain, occupying almost the entire region (Figure 1E,F).

Immunohistochemical analysis using coronal sections was consistent with the results in flat whole-mount preparation; both Robo1 and Robo2 were expressed strongly by longitudinal axons and weakly by circumferential axons crossing the midline at E14 (Figure S2A,C,D,F in Additional file 3).

Taken together, these observations indicate that Robo1 and Robo2 are largely expressed by longitudinally extending fibers during the stage when CF axons cross the midline and extend longitudinally.

### Expression of Robo1 and Robo2 mRNAs in the CP

**Figure 2:**
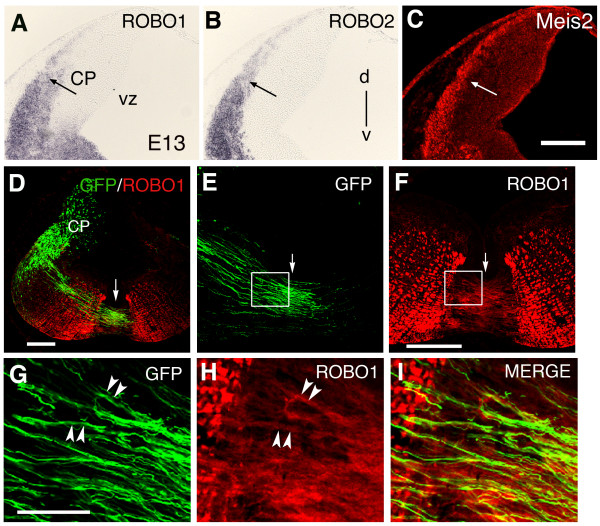
**(A,B) *In situ *hybridization for *Robo1 *(A) and *Robo2 *(B) in coronal sections of the cerebellar plate (CP).** Both *Robo1 *and *Robo2 *mRNAs are expressed in the region where deep cerebellar nucleus neurons should be located (arrows). d is dorsal and v is ventral. (C) Immunostaining, in an adjacent section, for Meis 2, a transcription factor known to be expressed in developing deep cerebellar neurons. (D) Coronal section of an E13 rostral hindbrain that was *in utero *electroporated at embryonic day 11 to introduce *egfp *plasmid into the vz and upper rhombic lip of the cerebellar plate. Axons can be seen to emanate from cell bodies located in the nuclear transitory zone and project ventrally to cross ventral midline. (E) *egfp*+ axons in the ventral midline region. (F) Robo1 immunostaining of the same section as in (E) shows numerous Robo1+ circumferential axons at the midline region. (G-I) Higher power and merged views of boxed areas in (E,F). At least a subset of *egfp*+ axons (arrowheads in G) expressed Robo1 (arrowheads in H; see also merged image in I). vz, ventricular zone. Arrows in (D-F) indicate ventral midline. Scale bar = 200 μm in (C), which applies to (A-C); 300 μm in (D); 300 μm in (E,F); 75 μm in (G-I).

The failure to detect Robo1 and Robo2 immunoreactivities in the CP prompted us to perform *in situ *hybridization experiments to examine the expression of mRNA of Robo1 and Robo2 in the cell soma of CF axons. As illustrated in Figure 2A,B, both *Robo1 *and *Robo2 *were expressed in the CP of E13 rat embryos. The signals were detected in the nuclear transitory zone, a region giving rise to the deep cerebellar nuclei [20]. Indeed, the region of mRNA signals partially overlapped with an immunoreactivity of the transcription factor Meis2, known as a marker for deep cerebellar nuclei cells [21] (Figure 2C). Similar results were obtained at E14 and E15 (data not shown), suggesting that deep cerebellar neurons continue to express mRNA of Robo1 and Robo2 at the time when CF axons cross the midline and extend longitudinally.

### Expression of Robo1 and Robo2 proteins on CF axons

Robo1 and Robo2 positive axons observed in the midline region partially overlapped with TAG-1 positive commissural axons (Additional file 3), supporting the notion that CF axons express Robo proteins. To directly examine whether axons originating from the deep cerebellar neurons express Robo1 or Robo2, we labelled progenitors of deep cerebellar neurons by *in utero *electroporation. For this, *egfp *plasmid was introduced into the ventricular zone of the CP using E11 mouse embryos and enhanced green fluorescent protein (EGFP) fluorescence was observed in coronal sections of the E13 mouse hindbrain (note that E13 mouse roughly corresponds to E15 rat). We found that EGFP-labelled axons emanating from the CP extended circumferentially toward the ventral midline and crossed it (Figure 2D–F). Immunostaining for Robo1 demonstrated that at least a subset of these axons express Robo1 protein near the midline (Figure 2G–I). Robo2 also appeared to be expressed by CF axons, although its expression was somewhat ambiguous (data not shown). These findings together suggest that Robo1 protein, and possibly Robo2 protein as well, are expressed on CF axons near the midline, raising the possibility that these proteins are involved in midline crossing of CF axons.

### Robo1 and Robo2 immunoreactivities are segregated from CF axon trajectories when these axons extend longitudinally

In *Drosophila *as well as in the rodent spinal cord, expression of Robo proteins appears to be up-regulated in commissural axons after midline crossing [11,14], which is thought to help them quit the midline and navigate longitudinally after midline crossing. To examine the possibility that Robo expression is also up-regulated in the hindbrain, we examined the expression of Robo1 at E16, when CF axons have crossed the midline and extend longitudinally [5]. To identify CF axons, 3,3'-dioctadecyloxacarbocyanine perchlorate (DiO) was injected into the CP (Figure S3A in Additional file 4). As can be seen in Figures S3B,D in Additional file 4, DiO-labelled fibres in the transverse section ran at a distance from the pial surface of the neural tube near the ventral midline. This tendency was retained after they had made a longitudinal turn (Figure S3E,G in Additional file 4). Near the region of the longitudinal turn, Robo1 immunoreactivity was clearly seen (Figure S3F,G in Additional file 4). However, its expression level was the highest near the pial surface (Figure S3C,F in Additional file 4), and barely detectable at the depth of the hindbrain where most CF axons were observed (Figure S3E,G in Additional file 4). Thus, contrary to our expectation, no or undetectable levels of Robo1 were expressed on CF axons in E16 rat embryos.

Similar results were obtained when the distribution of Robo2 immunoreactivity was compared with that of CF axon trajectories at E16 (Figure S4A in Additional file 5). As demonstrated in the previous section, DiO-labelled fibres ran at a distance from the pial surface of the neural tube in the ventral midline (Figure S4B in Additional file 5) and began to extend longitudinally at a distance from the midline (Figure S4E in Additional file 5). However, at this position along the dorsoventral axis, we could not observe immunoreactivities for Robo2 (Figure S4C in Additional file 5). Likewise, at this position along the mediolateral axis, we could not observe immunoreactivities for Robo2 (Figure S4F in Additional file 5), which are located more laterally. Thus, similar to Robo1, Robo2 expression in CF axons could not be detected at E16, although this does not preclude the possibility that these proteins are expressed at low levels.

### Trajectories of CF axons in Robo1/2 double knockout mice

**Figure 3:**
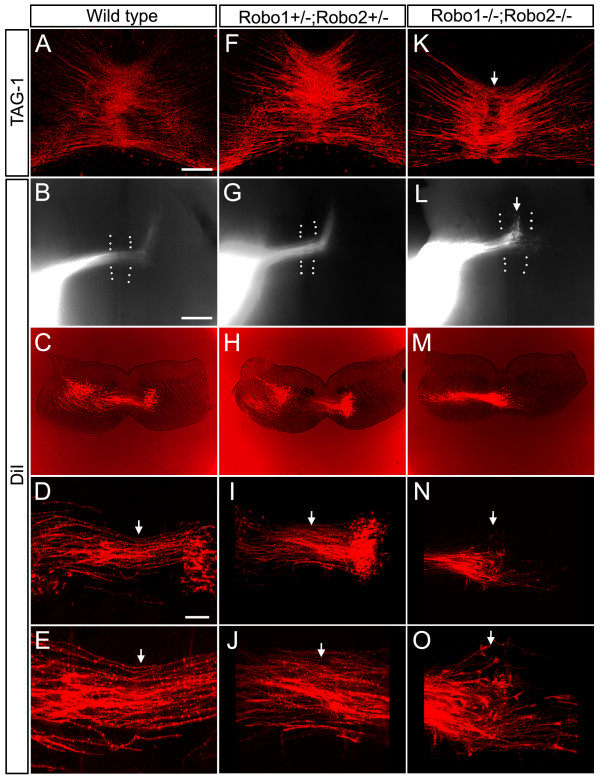
**Midline crossing errors in *Robo1*/*2 *double knockout mice**. (A,F,K) TAG-1 immunostained coronal sections of a wild-type (A), heterozygous (F) and homozygous mouse (K); dorsal is to the top. (B,G,L) DiI (1,1'-dioctadecyl-3,3,3',3'-tetramethylindocarbocyanine perchlorate) labelled flat mount preparations from a wild-type (B), heterozygous (G) and homozygous mouse (L). Dotted lines represent the approximate border of the floor plate; rostral is to the top. (C-E,H-J,M-O) Fluorescence micrographs of DiI-stained preparations. (D,I,N) Higher magnifications of (C,H,M), respectively. In (C,H,M), weak bright illumination is applied to show the contour of the sections. White arrows in (D,E,I,J,N,O) point to the midline. Note that TAG-1 immunoreactivity is weaker in the midline region of the double knockout mice (arrow in K). In DiI-stained preparations, labelled fibres stall near the midline (arrows in L,N,O). Dorsal is to the top. The bar in (A) is 150 μm and also applies to (F,K); bar in (B) is 400 μm and also applies to (C,G,H,L,M); bar in (D) is 200 μm and also applies to (I,N).

The expression of Robo1 and Robo2 on CF axons during midline crossing raises the possibility that Robo1, Robo2 or both are involved in midline crossing of CF axons. If this is the case, removal of Robo1 and 2 should cause pathfinding errors of CF axons in midline crossing. To test this, we first examined the trajectories of CF axons in *Robo1*/*2 *double knockout mice on E13 coronal sections immunostained for TAG-1. In coronal sections of wild-type and heterozygous (*Robo1*^+/-^; *Robo2*^+/-^) mice hindbrains at the level of CF axon decussation, TAG-1 immunoreactivity was found in circumferentially growing axons coursing not only superficially but also within the deep region where CF axons should reside (Figure 3A,F). In the midline region, no interruption of stained profiles was found, indicating that TAG-1 continues to be expressed in axons undergoing midline crossing, perhaps only being switched off when axons exit the FP. In contrast, in homozygous (*Robo1*^-/-^*;Robo2*^-/-^) mutants, TAG-1 staining appeared much weaker around the midline, leaving a dark strip in the middle (Figure 3K, arrow). Similar results were obtained by staining with anti-Rig-1 (data not shown), which stains commissural axons in a pattern similar to TAG-1 (see below). The most straightforward interpretation of these results is that CF axons stall at the FP, with most failing to cross the midline. Alternatively, it is possible that there is no change in CF trajectory but rather TAG-1 (or Rig-1) expression is down-regulated before CF axons enter the midline in the double knockout mice.

To distinguish these two possibilities, we prepared E14 mouse whole-mount preparations and implanted a small crystal of 1,1'-dioctadecyl-3,3,3',3'-tetramethylindocarbocyanine perchlorate (DiI) into the CP as above. In wild-type and heterozygous embryos, most DiI-labelled axons had crossed the midline and started to turn longitudinally (Figure 3B,G; n = 4/4 in each case of wild-type and heterozygous). In contrast, in four out of six samples from double knockout mice, tips of almost all DiI-labelled axons were found within the midline region (Figure 3L, arrow), with the other two samples displaying milder midline-crossing defects. This phenotype was confirmed by observing coronal sections. While many labelled axons reached the contralateral side in wild-type and heterozygous mice (Figure 3C–E,H–J; n = 3 in each case), most labelled axons appeared to be stalled within the FP in the double knockout mice (Figure 3M–O, see arrows in N and O; n = 4). It is noteworthy that the midline-crossing defect observed here is more marked than in the spinal cord, where only a minor population of commissural axons showed aberrant behaviours [13].

Taken together, these findings indicate that CF axons tend to stall within the midline region in the absence of Robo1 and Robo2.

### Expression of Rig-1 in flat, whole-mount rostral hindbrain preparations

**Figure 4:**
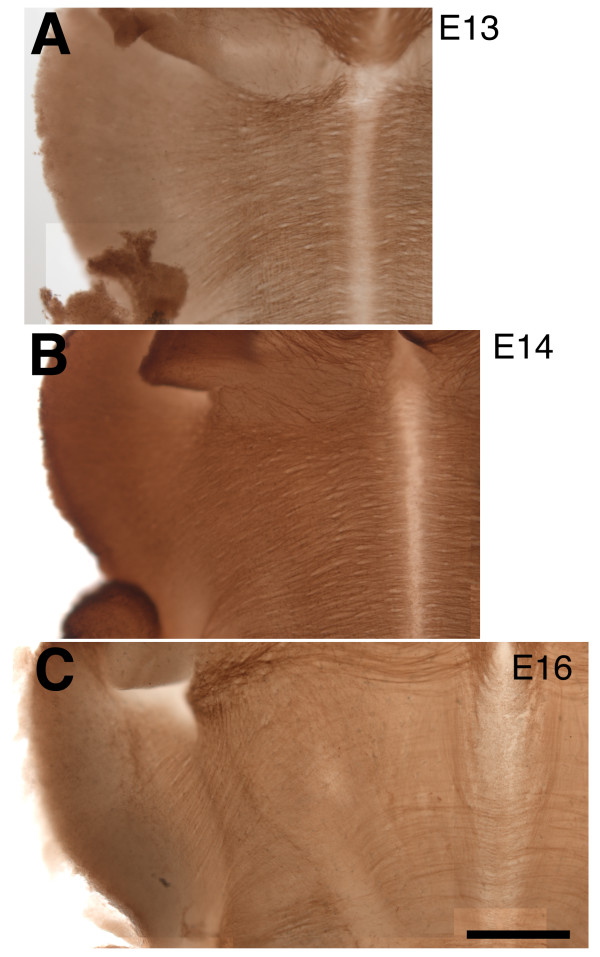
**Distribution of Rig-1 proteins in the cerebellofugal axonal pathway in a flat, whole-mount preparation**. (A-C) Embryonic day (E)13, E14 and E16 rat preparations, respectively. Note that at both E13 and E14, circumferentially growing axons are stained except at the ventral midline region. (C) Immunoreactivity decreased at E16. Scale bar = 200 μm.

In contrast to Robo1 and Robo2, high-level Rig-1 expression was found in circumferentially growing axons. Figure 4 illustrates immunostaining of Rig-1 in rat flat, whole-mount preparations. At E13 and E14, many Rig-1 immunoreactive fibres were observed to run circumferentially except in the CP. Immunoreactivity was reduced in the ventral midline region. A similar staining pattern was observed at E16, although the intensity of immunoreactivity was significantly reduced (Figure 4C). The pattern of Rig-1 staining on flat, whole-mount rostral hindbrains suggests the strong possibility that Rig-1 could be expressed in circumferentially growing CF axons.

### Expression of Rig-1 by CF axons

**Figure 5:**
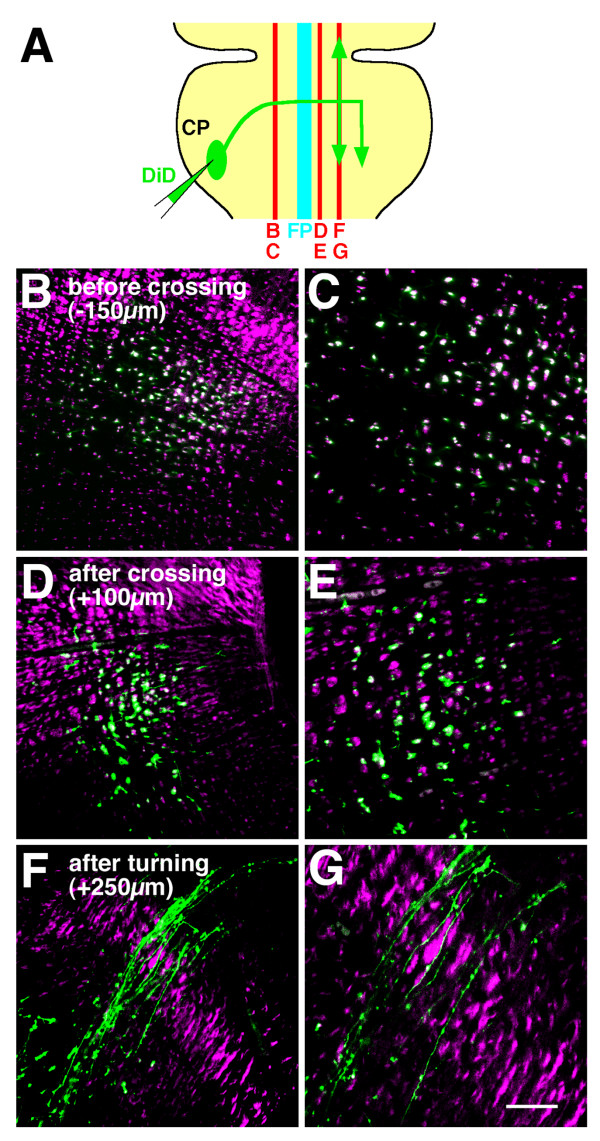
**Comparison of cerebellofugal (CF) axon trajectories with Rig-1 immunoreactive axons**. (A) A schematic view showing DiD (1,1'-dioctadecyl-3,3,3',3'-tetramethylindodicarbocyanine, 4-chlorobenzenesulfonate salt)-labelled CF axons (green) and the relative positions of the parasagittal sections shown in (B-G) (indicated by red lines and the letters beneath). (B-G) DiD-labelled CF axons (green) and Rig-1 immunoreactivity (purple) in parasagittal sections of embryonic day 16 preparations. (C,E,G) Magnifications of (B,D,F), respectively. Parasagittal sections were located at a distance of 150 μm (B,C) from the floor plate on the ipsilateral side, and 100 μm (D,E) and 250 μm (F,G) on the contralateral side relative to the DiD injection site. Scale bar = 100 μm for (B,D,F); 50 μm for (C,E,G).

To ensure expression of Rig-1 by CF axons, parasagittal sections of E16 rat hindbrain with the CF axons anterogradely labelled with 1,1'-dioctadecyl-3,3,3',3'-tetramethylindodicarbocyanine, 4-chlorobenzenesulfonate salt (DiD), were subjected to Rig-1 immunohistochemistry. We observed a high degree of co-localization of DiD-labelled, circumferentially growing CF axons with Rig-1-positive axons on the sections ipsilateral to the DiD injection site (Figure 5B,C). However, this co-localization was dramatically decreased on the contralateral side (Figure 5D–G). After CF axons had crossed the midline but before they took a longitudinal path, there still remained a small degree of co-localization (Figure 5D,E). However, almost all CF axons were Rig-1 negative after turning longitudinally (Figure 5F,G). Taken together, these data show that Rig-1 is expressed on pre-crossing CF axons, but its expression is markedly decreased after midline crossing and becomes undetectable after CF axons turn longitudinally. Expression of Rig-1 by (pre-crossing) CF axons was also supported by double staining of whole-mount preparations with anti-Rig-1 and anti-TAG-1 antibodies (data not shown).

### Attraction of Rig-1-positive axons by the floor plate

**Figure 6:**
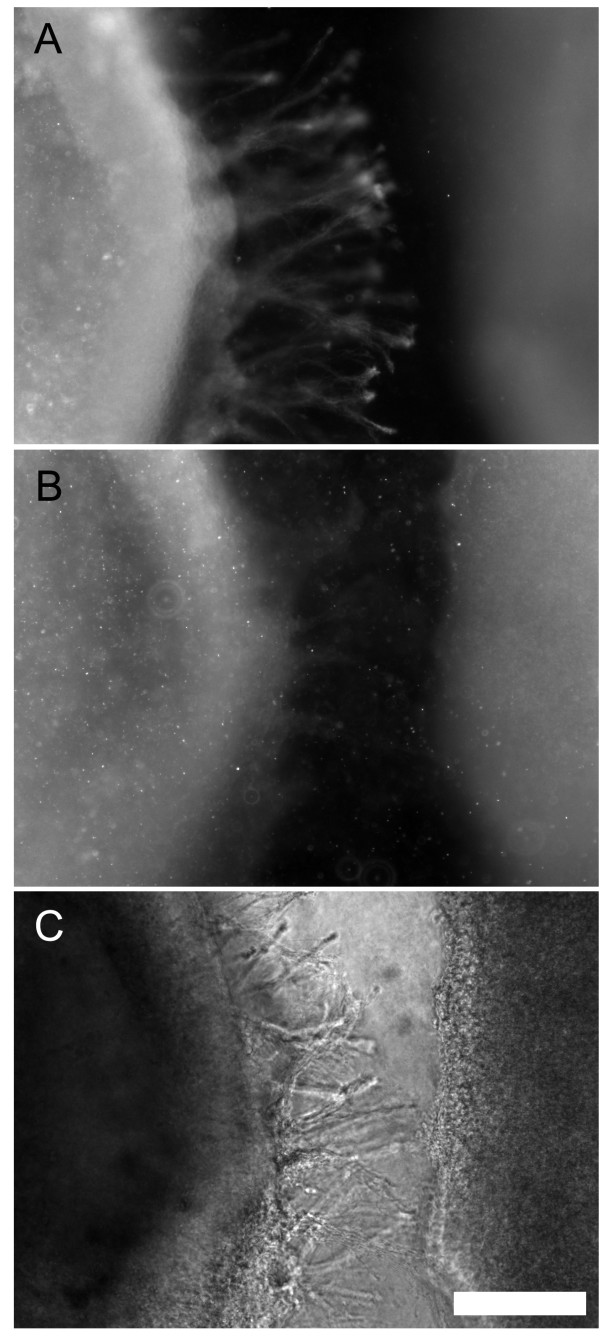
**Neurites elicited in co-culture with the floor plate (FP) express Rig-1**. (A) Fluorescence micrograph of a co-culture of a cerebellar plate with an FP explant. The culture was immunostained for anti-Rig-1 antibody after culture. Note that extensive growth of Rig-1-positive axons occur towards the FP. (B) Similar to (A) but a control in which Rig-1 antibody pre-absorbed by recombinant Rig-1eFc was applied. (C) Phase contrast micrograph of the field that corresponds to (B), showing extensive growth of neurites. Scale bar = 200 μm.

CF axons are attracted by the FP and by Netrin-1 *in vitro *[5,22]. Therefore, we performed collagen-gel cultures and analysed the expression of Rig-1 by CF axons elicited in a co-culture with the FP to further ensure Rig-1 expression by CF axons. In accordance with previous reports [5,22], extensive growth of neurites occurred from CP explants on the side facing the FP. Immunostaining with the Rig-1 antibody demonstrated that these neurites express Rig-1 protein (Figure 6A). In control experiments, in which pre-absorption with Rig-1 antigen was performed, virtually no neurite was stained (Figure 6B). These results further support our conclusion that CF axons express Rig-1.

### Trajectories of CF axons in *Rig-1 *knockout mice

**Figure 7:**
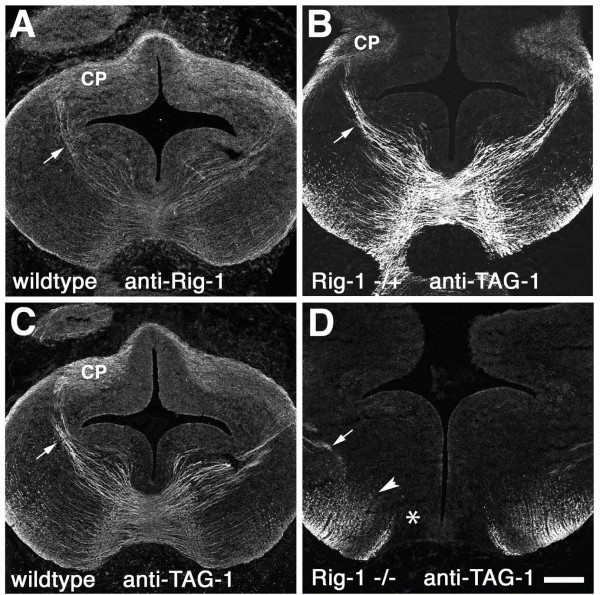
**Failure of cerebellofugal (CF) axons crossing the midline in *Rig-1 *knockout mice**. (A-D) Transverse sections including the circumferential CF axon pathway were prepared from embryonic day 13 wild-type (A,C), *Rig-1 *heterozygous (B) and homozygous (D) mice and stained with anti-Rig-1 (A) or anti-TAG-1 (B-D) antibodies. Both Rig-1 (A) and TAG-1 (B) were expressed by CF axons (arrows) running from the cerebellar plate (CP) toward the floor plate (FP) in wild-type mice. TAG-1 positive axons were not observed around the FP in *Rig-1 *homozygotes (D), indicating that commissural axons, including CF axons (arrow), fail to cross the FP. Arrowhead in D indicates TAG-1 positive axons coursing longitudinally. No stained axons can be seen near the midline (asterisk). Dorsal side is to the top in all panels. Scale bar in (D) is 200 μm and applies to all panels.

Analysis of *Rig-1 *knockout mice showed that CF axons fail to cross the ventral midline. In wild-type animals, many Rig-1-positive axons ran circumferentially toward the FP while axons that appear to be CF axons were clearly visualized (Figure 7A, arrow). These axons departed from the CP and headed toward the midline and coursed deep into the pial surface, consistent with the results of lipophilic-dye tracing experiments (Figure 5; n = 2/2). Immunostaining with an antibody against TAG-1 provided a pattern of staining almost identical to that of anti-Rig1 (Figure 7B,C) in wild-type as well as heterozygous mice (n = 2/2 for each). In contrast, in *Rig-1 *knockout mice, although TAG-1-positive CF axon-like fibres were observed, their circumferential trajectory was terminated before they approached the midline (Figure 7D, arrow; n = 2/2). Instead, clusters of immunoreactivities that appeared to be cross-sections of axons (Figure 7D, arrowhead) occupied the ventral part of the hindbrain, suggesting that CF axons course longitudinally. Moreover, no immunoreactivity was observed near the ventral midline (Figure 7D, asterisk).

The disappearance of commissural axons near the midline region appeared to be caused by the aberrant trajectory of these axons. TAG-1 immunostaining of the whole-mount preparation of the neural tube illustrated that commissural axons appeared to have changed their trajectory from circumferential to longitudinal, at a distance from the FP in *Rig-1 *knockout embryos (Additional file 6), suggesting that these axons turned longitudinally instead of approaching the FP. These results indicate that Rig-1 is required for CF axons to enter the midline, as is the case in the spinal cord [14].

## Discussion

### Expression of Robo proteins on CF axons

**Figure 8:**
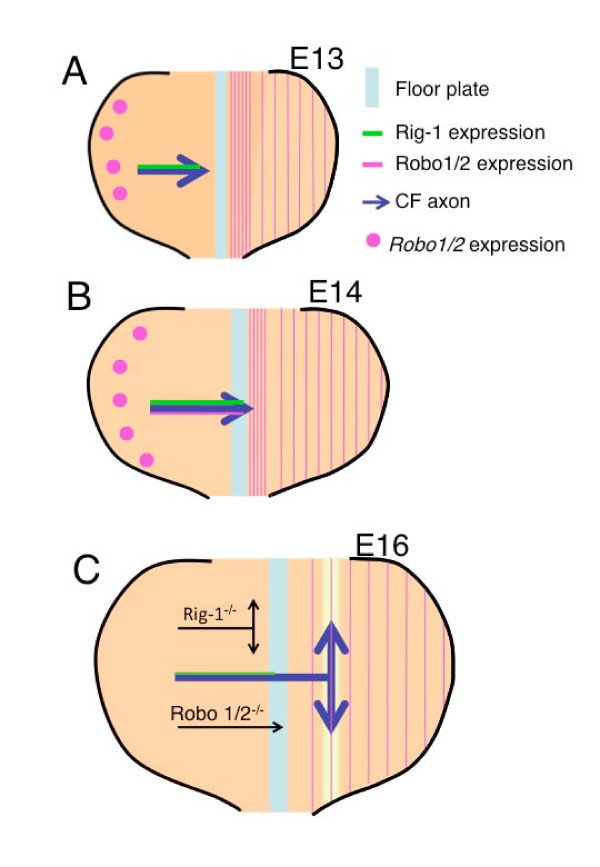
**Summary of the present results**. Schematics showing growing cerebellofugal (CF) axons in open book preparations of the rat hindbrain. (A) At embryonic day (E)13, CF axons extend circumferentially and have not arrived at the ventral midline floor plate, while Robo1 and Robo2 immunoreactivities are largely confined to the longitudinal axons. (B) At E14, CF axons reach the floor plate. At this stage, immunoreactivity for Robo1 and 2 can be detected for CF axons in the midline region. (C) At E16, CF axons extend longitudinally but weak or no expression of Robo1, Robo2 or Rig-1 can be detected on the post-crossing portion of CF axons. In the *Rig-1 *mutant, CF axons fail to reach the midline and extend longitudinally on the ipsilateral side. Circles represent Robo1/2 mRNA expression, arrows CF axon trajectories, green lines Rig-1, and purple lines Robo1/2. The yellow strip represents distribution of a presumptive favourable cue. In *Robo1*/*2 *double knockout mice, many CF axons stall at the floor plate. mRNA expression has not been tested at E16. E represents embryonic day in rat.

Immunohistochemical analyses of flat, whole-mount preparations with specific antibodies revealed that three Robo proteins, Robo1, Robo2 and Rig-1, show distinct expression patterns in the developing hindbrain of the rat. Robo1 and Robo2 are mainly expressed by axons longitudinally growing near the FP and at the intermediate level along the circumferential axis, respectively, while Rig-1 is expressed by circumferentially growing axons (Figure 8A,B). Analyses of coronal sections, however, revealed that both Robo1 and Robo2 seem to be expressed in CF axons near the midline (Figure 8B, horizontal purple line). Consistent with these, expressions of Robo1 and Robo2 mRNAs were observed in the CP before and at the stage of CF axon midline crossing (Figure 8A,B, purple dots). At a later stage when CF axons elongate longitudinally, the region occupied by Robo1 or Robo2 immunoreactive axons coursing either circumferentially or longitudinally was, for the most part, segregated from CF axon trajectories, failing to support the notion that these proteins are up-regulated after midline crossing unlike the case of spinal cord.

The Robo1 as well as Robo2 immunoreactive longitudinal axons were observed in the hindbrain at E13 before CF axons reached the midline. There were also a small number of midline crossing axons (Figure 1D, arrow). These results raise the possibility that axons other than CF axons also express Robo1 and Robo2 in the rostral hindbrain. In the hindbrain, the earliest commissural axons originate from neurons located near the midline and cross the midline at around E10 in mouse (Y Furukawa, K Yamauchi and F Murakami, unpublished observations). Moreover, *Robo1 *and *Robo2 *are also expressed in the basal plate (data not shown). Therefore, it is possible that Robo1- and Robo2-immunoreactive longitudinal axons originate from these early developing commissural axons whose cell bodies are located near the ventral midline.

Rig-1 was highly expressed in a large population of CF axons before they crossed the FP, but not in the post-crossing portion of CF axons (Figure 8, green). Expression of Rig-1 in the pre-crossing portion of commissural axons was also indicated in the spinal cord by comparing Rig-1 expression and TAG-1 expression [14]. In the present study, we have directly shown that post-crossing down-regulation of Rig-1 expression in CF axons takes place by double labelling of CF axons with an anti Rig-1 antibody and anterogradely labelling with a lipophilic dye.

### Role of Robo1 and Robo2 in the guidance of CF axons

The present results demonstrate that Robo1, Robo2 or both are required for CF axons in crossing the midline and exiting from it. Both Robo1 and Robo2 appeared to be expressed in circumferential axons in the midline region when CF axons cross the ventral midline. The finding that *Robo1 and Robo2 *mRNAs were expressed in the CP at this stage and before CF axons cross the midline supports the idea that Robo1 and Robo2 are expressed in midline-crossing CF axons. In *Robo1*/*2 *double knockout mice, many CF axons stalled at the midline region. Taken together, these results support the notion that Robo1 and Robo2 play crucial roles in midline crossing of CF axons.

However, the present double labelling experiments failed to show immunoreactivities of either Robo1 or Robo2 in CF axons at the stages when they initiate longitudinal growth (Figure 8C). This contrasts with observations in the spinal cord where expression of Robo1 and Robo2 appears to be up-regulated in the longitudinal portion of post-crossing commissural axons [13]. Thus, while pathfinding of spinal commissural axons is consistent with the model describing augmented repulsion by FP-derived repellents, including Slit, after axons cross the midline [9], this does not seem to hold true for hindbrain commissural axons. In the rostral hindbrain, where CF axons execute a longitudinal turn, the motor column, which expresses Slit in the spinal cord [23] and is thought to contribute to post-crossing navigation of commissural axons [10], does not exist. Thus, the model that post-crossing axons are propelled into a longitudinal pathway by Slit expressed in the midline and ventral spinal cord [10] does not seem to hold true for post-crossing navigation of CF axons (see below).

The midline-crossing phenotype in *Robo1*/*2 *double knockout mice reported here may be explained by assuming that activation of Robo1 or Robo2 is required to silence the attractive effect of Netrin-1, as was proposed previously based on *in vitro *experiments using *Xenopus *neurons [9], thus promoting midline exit. In *Robo1*/*2 *double knockouts, the attractive effect by Netrin-1 might not be attenuated, impeding the departure of CF axons from the midline.

From our results, we cannot determine which of the two Robos plays key roles in CF axon midline crossing. Robo 1 and Robo 2 appear to cooperatively guide axons of the lateral olfactory tract [17] and forebrain major axonal tracts [24]. However, the previous finding that Robo1 regulates midline crossing of the spinal commissural axons [13] favours the view that Robo1 is crucial for the phenotype observed in this study. A similar finding was recently reported for the formation of the corpus callosum, where Robo1 but not Robo2 is expressed and *Robo1 *knockout mice display malformations [16]. The present immunohistochemical observation that Robo1 was unequivocally expressed by CF axons strongly supports the role of Robo1, although involvement of Robo2 cannot be precluded.

### Rig-1 is involved in the guidance of CF axons

The failure of CF axons in *Rig-1 *knockout mice to cross the midline clearly indicates that Rig-1 is required for CF axon approach to the midline. Consistent with this, we found that Rig-1 was expressed by CF axons before midline crossing and was dramatically reduced after midline crossing (Figures 5 and 8). Similar expression patterns for Rig-1 and phenotype in *Rig-1 *knockout mice have been observed in the spinal cord [14], suggesting that a common mechanism operates for midline crossing between the spinal cord and the hindbrain. Recently, two isoforms of Rig-1 (Robo3) with distinct cytoplasmic carboxy-terminal regions have been found, one of which, Robo3.1, is expressed on the pre-crossing portion and another, Robo3.2, on the post-crossing portion of spinal commissural axons [15]. Since our antibody against ectodomain recognizes both isoforms, post-crossing CF axons do not seem to express either isoform.

The phenotype of midline crossing failure observed in the spinal cord can be partially rescued by removing *Slit1 *and *Slit2 *or *Robo1*, suggesting that removal of Rig-1 up-regulates commissural axon sensitivity to midline Slits, preventing midline crossing [14]. Consistent with this, a failure of neurite outgrowth from the dorsal spinal cord explant of *Rig-1 *knockout mice towards a FP explant turns into robust growth of neurites in the presence of Robo2 ectodomain proteins [14]. Our results showing that CF axons had midline crossing defects similar to spinal commissural axons suggest that Rig-1 also plays a crucial role in midline crossing of hindbrain commissural axons and support the possibility that Rig-1 plays a global role in commissural axon guidance at all axial levels.

It is noteworthy that all commissural axons as visualized by TAG-1 staining were affected in the Rig-1 mutant (Figure 7; Additional file 6). This indicates that Rig-1 plays a crucial role in midline crossing of various types of axons in the hindbrain.

### Relationship between Rig-1 and Robo1 function

The present results that up-regulation of either Robo1 or Robo2 was not observed following midline crossing raise the possibility that down-regulation of Rig-1 from CF axons regulates midline crossing by a mechanism that is independent of Slit-Robo signalling. One possibility to explain the behaviour of post-crossing axons without assuming a Rig-1-Robo1 interaction is gain of responsiveness to a favourable cue. We have previously proposed a model that CF axons develop a gain of responsiveness to a favourable cue to explain the behaviour of the longitudinal turn in CF axons [25]. CF axons become sensitive to a longitudinally aligned putative cue in the basal plate (Figure 8C, yellow strip) after ventral midline crossing, allowing them to make a longitudinal turn [25]. This idea tempts us to hypothesize that pre-crossing CF axons may normally be unable to respond to this presumptive cue expressed in the basal plate due to the presence of Rig-1. However, down-regulation of Rig-1 in post-crossing CF axons abolishes the inhibition (Figure 4). In *Rig-1 *mutants, CF axons turn longitudinally on the ipsilateral side because the absence of Rig-1 should enable them to recognize the basal plate cue there. The validity of this model awaits further studies on this hypothesized basal plate cue.

## Conclusion

We provide evidence that Rig-1 is expressed by CF axons and is required for their approach to the midline, suggesting that Rig-1 plays a critical role in ventral midline crossing of commissural axons at all axial levels. Moreover, disruption of CF axon pathfinding in *Robo1*/*2 *double knockout mice demonstrates that Robo1, Robo2 or both contribute to the midline crossing of these axons. However, the failure to detect high level Robo1 and Robo2 expression on these axons during the longitudinal growth of CF axons suggests that expression of Robo1 and Robo2 are not required for longitudinal navigation of CF axons. Nevertheless, CF axons share some guidance mechanisms with spinal commissural axons as both: are attracted by midline Netrin-1 [3,4]; express TAG-1 before midline crossing; change responsiveness to FP cues [8,10]; and show a sharp turn from the circumferential to longitudinal axis after midline crossing [25]. Our results illustrate that although common mechanisms operate for midline crossing of commissural axons at different axial levels, some variation does exist in post-crossing navigation.

## Materials and methods

### Preparation of recombinant Robo-Fc fusion proteins

cDNAs encoding ectodomains of rat Robo1 (amino acids 1–892), rat Robo2 (amino acids1–855) and mouse Rig-1 (amino acids1–864) were obtained by PCR using E15 rat or E14 mouse brain cDNA as a template. They were subcloned into a pCAG/Fc vector, which was modified from pCAGGS, a mammalian expression vector under the control of CAG promoter [26], to enable expression of a fusion protein connected with the Factor Xa digestion site ('IEGR') and the human IgG1 Fc region. When COS-7 cells were transiently transfected with these vectors, the Fc chimeric protein of the Robo1 ectodomain (Robo1eFc) and that of the Robo2 ectodomain (Robo2eFc) were secreted into the culture medium. However, since the Fc chimeric protein of the Rig-1 ectodomain (Rig-1eFc) was not secreted, the Rig-1eFc vector was modified to express secreted Rig-1eFc protein by replacing the signal peptide of Rig-1 (amino acids 1–18) with that of human IgG1 ('MDWTWRILFLVAAATGAHS').

The chimeric proteins were prepared in COS-7 cells by the DEAE-dextran transfection method according to the manufacturer's instructions (ProFection Mammalian Transfection Systems, Promega, Madison, WI, USA) with some modifications. In detail, 7.5 × 10^5 ^COS-7 cells were plated on 100 mm culture plates. Next day, the cells were washed twice with phosphate-buffered saline (PBS; Dulbecco's PBS(-), pH 7.4, Nissui, Tokyo, Japan). They were then incubated with 1 ml of DNA-DEAE-Dextran mixture (5 μg of expression vector, 0.5 mg/ml of DEAE-Dextran) in PBS for 30 minutes, followed by 6 ml of Dulbecco's modified Eagles medium (DMEM) containing 80 μM chloroquine for 5 h. The cells were further cultured overnight by replacing the medium with DMEM/10% fetal bovine serum (FBS). The cells were washed three times with DMEM and incubated with DMEM/F12 for 3 days. The chimeric proteins were purified from culture supernatant using a Protein A Sepharose column (Amersham Biosciences, Piscataway, NJ, USA).

### Antibody production

Antibodies against Robo1, Robo2 and Rig-1 were produced by immunizing rabbits with an endermic injection of the recombinant Robo1eFc, Robo2eFc and Rig-1eFc proteins, respectively (QIAGEN, Hilden, Germany). Total IgG fraction was purified from serum by protein A-sepharose chromatography. The antibody against Meis2 was produced by immunizing rabbits with an endermic injection of synthesized peptide corresponding to the most amino-terminal 15 amino acid sequence of mouse Meis2 (QIAGEN).

### Animals

Embryonic rats used in this study were timed-pregnant Wistar rats (Nihon-SLC, Shizuoka, Japan). The day on which the plug was detected was designated as E0. Embryos were removed from pregnant rats that had been deeply anesthetized with sodium pentobarbitone (Nembutal, Abbott, North Chicago, IL; 50 mg/kg body weight). All experiments were conducted in compliance with the Guidelines for Use of Laboratory Animals of the National Institute for Basic Biology and Osaka University.

*Rig-1*-deficient mice were processed as previously described [14]. Generation of *Robo1*/*Robo2 *double knockout mice has been described previously [15].

### Western blot analysis

One hundred nanograms of recombinant protein was used. In some cases, the protein was digested with Factor Xa (New England Biolabs Inc., Beverly, MA, USA) at 23°C overnight. The protein was separated by 5–20% gradient SDS-PAGE and blotted onto a nitrocellulose membrane (BA-S85, Schleicher&Schuell, Dassel, Germany) by a semidry blotting system (AE-6677, ATTO, Tokyo, Japan).

The membrane was incubated at room temperature with 5% skim milk in Tris-buffered saline containing 0.05% Tween20 (TTBS) for 30 minutes, followed by a primary antibody in TTBS (2 μg/ml for Robo1 or Robo2, 5 μg/ml for Rig-1) or alkaline-phosphatase (AP) conjugated anti-human IgG-Fc antibody (Jackson ImmunoResearch, West Grove, PA, U.S.A) overnight. They were washed three times with TTBS for 5 minutes. When Robo antibodies were used, membranes were further incubated with AP conjugated anti-rabbit IgG (Roche, Basel, Switzerland) for 2 h. After washing three times with TTBS for 5 minutes, membranes were incubated with AP buffer (100 mM Tris-HCl, pH 9.5, 100 mM NaCl, 50 mM MgCl_2_), and immersed in NBT/BCIP solution (NBT, 0.375 mg/ml; BCIP, 0.188 mg/ml; Roche) in AP buffer to develop colour signals.

### Immunohistochemistry

The procedures for preparing flat, whole-mount hindbrain followed those described in [5] with some modifications. E13–E16 rats were removed and the hindbrain was dissected. After the hindbrain was cut along the dorsal midline, meninges were removed and the remainder of the brain was opened and flat, whole-mounted with the ventricular side down. All dissection procedures were performed in cold PBS solution. The brain was then fixed by immersion in 4% paraformaldehyde (PFA) in 0.1 M phosphate buffer (PB; pH. 7.4) and stored at 4°C for several days. All procedures were done at room temperature thereafter. The preparations were incubated in a solution of 3% hydrogen peroxide in methanol for 30 minutes to inhibit endogenous peroxidase activity and washed three times with Tris-buffered saline, pH 7.4, containing 2% Triton X-100 (TBS-T) for 10 minutes. The preparations were blocked with 10% normal goat serum in TBS-T for 30 minutes and treated with primary antibodies (2 μg/ml against Robo1 or Robo2; 5 μg/ml against Rig-1) diluted in TBS-T containing 1% normal goat serum overnight. When the specificity of the primary antibodies was examined, preparations were pre-absorbed overnight with recombinant protein at a ratio of 100:1. After preparations were washed three times with TBS-T for 20 minutes, they were incubated with biotin conjugated goat anti-rabbit IgG antibody (BA-1000, 1:200; Vector Laboratories, Burlingame, CA, USA) as the secondary antibody for 2 h. After three washes with TBS-T for 20 minutes and with TBS for 20 minutes, the preparations were incubated in avidin-biotin peroxidase complex (ABC; Vector Vectastain ABC Elite kit, diluted 1:100 in TBS) for 2 h. After three washes with TBS for 20 minutes, the preparations were incubated for 40 minutes in diaminobenzidine tetrahydrochloride (0.1% in TBS) with 0.002% H_2_O_2 _and 0.04% NiSO_4_.

For frozen sections, brains were removed from the pregnant rat and fixed in 4% PFA/0.1 M PB. The fixed brains were immersed in 20% sucrose/0.1 M PB overnight and embedded in OCT compound (Sakura Finetechnical Co., Ltd, Tokyo, Japan). Coronal sections were cut at 16 μm thickness with a cryostat (Microm, HM500M, Zeiss, Jena, Germany) and mounted on slides coated with poly-L-lysine (Matsunami Glass Inc., Ltd, Osaka, Japan). For immunohistochemistry, almost all procedures were the same as above, but with some modifications. Sections were incubated in TBS containing 0.2% Triton X-100 instead of 2% Triton X-100. In all washing steps, the time for incubation was 10 minutes.

For immunohistochemistry of *Rig-1*-deficient mice and *Robo1*/*2 *double knockout mice, E13 embryos were fixed as previously described [11] and cut into 20 μm thick transverse sections. The sections were reacted with the anti-Rig-1 or anti-TAG-1 (4D7, Developmental Studies Hybridoma Bank, no dilution) antibodies, then with Cy3-conjugated anti-rabbit or anti-mouse IgG antibodies (Jackson ImmunoResearch). Whole-mount preparations of *Rig-1 *deficient mice were stained for TAG-1 as described previously [14].

### *In situ *hybridization

cDNAs encoding ectodomains of rat Robo1 and Robo2 subcloned into pBluscriptSK were used to prepare sense and antisence RNA probes. The method for *in situ *hybridization followed Hasegawa *et al*. [27] except that treatment with proteinase K was extended to 15 minutes.

### *In utero *electroporation

The method of *in utero *electroporation will be detailed elsewhere. In brief, 2 μl EGFP plasmid was injected into the fourth ventricle and was electroporated to the cerebellar ventricular surface and the upper rhombic lip of E11 C57BL6 mice embryos. For electroporation, five 50 ms pulses with an amplitude of 40 V were applied at 950 ms intervals. One to two days after the electroporation, the embryo was fixed and the hindbrain was dissected out for cryosectioning.

### Double labelling with DiO or DiD and fluorescence immunohistochemistry

Flat, whole-mount preparations were fixed in 4% PFA/0.1 M PB for 1 day at 4°C. Small crystals of the fluorescent tracer DiO (Invitrogen, Carlsbad, CA, USA) or DiD (Invitrogen, Eugene, OR, U.S.A.) were implanted into the CP, which is the primordium of the cerebellum. The preparations were kept in 4% PFA/0.1 M PB overnight at room temperature. Then the preparations were stored in PBS containing 0.1% EDTA for 2–4 days at room temperature to allow for dye diffusion. Afterwards, the preparations were embedded in 4% low-melting agarose (SIGMA Aldrich, Tokyo, Japan) followed by sectioning coronally or parasagittaly into 50 μm thick slices by a vibrating blade microtome (VT1000S, Leica Microsystems, Tokyo, Japan) in PBS followed by immunohistochemistry.

Fluorescence immunohistochemistry on these sections was performed essentially the same as immunostaining on flat-mount hindbrain (see above) with some modifications. The incubation with 3% hydrogen peroxide was omitted. All procedures were carried out in solutions without any detergent. And lastly, Cy3- or Alexa Fluor 594-conjugated streptavidin instead of avidin-biotin peroxidase complex was used for visualization.

Mouse embryos single labelled with DiI were treated similarly to double labelled ones.

### Explant culture preparations

E13–14 rat embryos were dissected in DMEM/F12 medium (Sigma, cat. no D-8900) with glucose (3.85 mg/ml). CP and FP explants were removed from longitudinally opened hindbrain using tungsten needles. After trimming, the explants were embedded together in collagen gels (separation < 500 μm). CP explants were co-cultured at 37°C in 5% CO_2 _for 24–48 h with FP explants. The culture medium was DMEM/F12 medium supplemented with 3.85 mg/ml glucose, N2 Supplement (Invitrogen, Grand Island, NY, catalogue no 17502-048) and 10% FBS. Explant cultures were fixed in 4% PFA/0.1 M PB for 6–12 h and observed with a phase contrast microscope. Following capture of phase contrast images, they were subjected to immunohistochemistry. For immunostaining, the explants were washed with PBS and permeated in PBS containing 0.2% TritonX-100 (0.2% PBST) followed by blocking in 10% normal goat serum for 1 h at room temperature. The explants were incubated with the anti-Rig-1 antibody (5 μg/ml) overnight at 4°C. After two washes each, for 30 minutes in 0.2% PBST, they were incubated in Cy3-conjugated anti-rabbit antibody (1:250; Jackson ImmunoResearch) for 2 h at room temperature.

### Image processing

Flat, whole-mount preparations were gently coverslipped in TBS. The preparations were then observed with a light microscope (BX60, Olympus, Tokyo, Japan) equipped with a CCD camera (HRc, AxioCAM, Zeiss or CoolSNAP HQ, Roper). For vibratome sections double labelled with DiO or DiD and Cy3-conjugated (or Alexa Fluor 594-conjugated) streptavidin, images were captured by a CCD camera (C4880-40-26A, Hamamatsu, Shizuoka, Japan) for DiO and confocal microscopy (MRC1024ES, BIORAD) for DiD. For phenotype analysis in *Rig1 *or *Robo1*/*Robo2 *mutants, vibratome sections with DiI labelling were imaged with a fluorescence microscope (BX60, Olympus, Tokyo, Japan) coupled with a CCD camera (AxioCAM, Zeiss). Fluorescent images of frozen sections were acquired by a scanning confocal microscope (TCS SP2 AOBS, Leica). The images were processed using Adobe Photoshop software (Adobe Systems, Mountain View, CA, USA).

## Abbreviations

AP: alkaline-phosphatase; CF: cerebellofugal; CP: cerebellar plate; DiD: 1,1'-dioctadecyl-3,3,3',3'-tetramethylindodicarbocyanine, 4-chlorobenzenesulfonate salt; DiI: 1,1'-dioctadecyl-3,3,3',3'-tetramethylindocarbocyanine perchlorate; DiO: 3,3'-dioctadecyloxacarbocyanine perchlorate; DMEM: Dulbecco's modified Eagles medium; E: embryonic day; EGFP: enhanced green fluorescent protein; FP: floor plate; PB: phosphate buffer; PBS: phosphate-buffered saline; PBST: PBS containing TritonX-100; PFA: paraformaldehyde; TBS-T: Tris-buffered saline; pH 7.4: containing 2% Triton X-100; TTBS: Tris-buffered saline containing 0.05% Tween20.

## Competing interests

The authors declare that they have no competing interests.

## Authors' contributions

TK prepared antigens of Robos, characterized antibodies and performed DiO-labelling and a part of the immunohistochemical experiments. AT conceived experiments on expression pattern of Robo proteins, and participated in antibody characterization and immunohistochemistry. TM carried out *in vitro *culture experiments, immunohistochemical analyses and *in situ *hybridization experiments. YZ developed the original idea, designed and performed DiD labelling experiments, the DiI analysis of *Robo1*/*2 *knockout mice and immunohistochemistry, and analyzed data. YH and YZ helped revise the manuscript. KM helped with immunohistochemistry of *Rig-1 *knockout mice. ZC prepared fixed embryos of *Robo1*/*Robo2 *double knockout mice. YT generated an antibody against Meis2. KY and MT designed and generated probes for *in situ *hybridization. HO and KN carried out *in utero *electroporation. FM participated in the overall design and coordination of the study and wrote the manuscript. All authors read and approved the final manuscript.

## Supplementary Material

Additional file 1 **Supplemental information.** Explanation of the procedures and specificity of the generated antibodies. Click here for file

Additional file 2 **Figure S1: procedures and specificity of the generated antibodies.** Specificity of rabbit anti-rat Robo1 and Robo2 antibodies and anti-mouse Rig-1 antibody. (A) Robo1eFc (lanes 1–3), Robo2eFc (lanes 4–6) and Rig-1eFc (lanes 7–9) proteins were separated by SDS-PAGE and blotted onto a nitrocellulose membrane. To identify Robo proteins, the membrane was reacted with an antibody against the human IgG1 Fc region (lanes 1, 4 and 7), IgG fractions purified from pre-immune sera (lanes 2, 5 and 8) or antisera (lanes 3, 6 and 9). Arrows indicate the molecular weight of the corresponding Robo-Fc fusion proteins. (B-D) Cy3 immunofluorescence of coronal sections from E14 rat hindbrain stained by antibodies raised against Robo1eFc, Robo2eFc and Rig-1eFc. (B'-D') Same as (B-D) but the antibodies were pre-adsorbed by excessive antigens. Scale bar = 200 μm. Click here for file

Additional file 3 **Figure S2: immunostaining of coronal sections with Robo1, Robo2 and TAG-1.** Robo1 and Robo2 immunoreactivies for circumferential axons in the midline region. (A-F) Immunostains for Robo1 (A,C), for Robo2 (D,F) and TAG-1 (B,E). Arrows indicate circumferentially growing axons in the midline. Asterisks show cross sections of longitudinally growing axons. Robo2 immunoreactive circumferential axons can be observed several distances from the ventral midline. These are unlikely to be post-crossing CF axons because CF axons make longitudinal turns in a region closer to the midline (Figure 7). Comparisons with immunostaining for TAG-1, which is expressed in hindbrain and spinal cord commissural axons before midline crossing [21], support the notion that CF axons express Robo1 as well as Robo2 (Figure 2B,E). Coronal sections of an E14 rat embryo. Scale bar = 150 μm in (A,B,D,E) and 75 μm in (C,F). Click here for file

Additional file 4 **Figure S3: segregation of Robo1 immunoreactivity and CF axon trajectory.** Comparison of CF axon trajectories with Robo1 immunoreactive axons. DiO was injected into the CP of E16 flat, whole-mounted hindbrain after fixation. After allowing for DiO diffusion, coronal sections (B-D) or parasagittal sections (E-G) of the brain were made and immunostained for Robo1. (A) Schematic showing the trajectory of DiO positive CF axons (green). Red lines indicate planes of the section that correspond to designated panels. (B-D) DiO-labelled axons and Robo1 immunoreactivity in a coronal section. Ipsi, ipsilateral; Contra, contralateral. (E-G) Robo1 immunoreactivity and ascending DiO-labelled axons in the parasagittal section. In both planes, the Robo1 immunoreactive region was located more superficially to the region where DiO-labelled axons were found. Scale bar = 200 μm. Click here for file

Additional file 5 **Figure S4: segregation of Robo2 immunoreactivity and CF axon trajectory.** Comparison of CF axon trajectories with Robo2 immunoreactive axons. E16 preparations were treated similarly to those in Figure S3 (Additional file 4) but immunostained for Robo2. (A) Schematic showing the trajectory of DiO positive fibres (green) and planes of the section (red). (B-D) DiO-labelled axons and Robo2 immunoreactivity in a coronal section. (D2) is a lower magnification view of (D1) showing Robo2 immunoreactive fibres running near the ventral surface (arrows). Ipsi, ipsilateral; Contra, contralateral. (E-G) DiO-labelled axons and Robo2 immunoreactivity in a parasagittal section. In both planes, Robo2 immunoreactivity, although weak, is located superficially to the region where DiO-labelled axons are found. White arrow in (F) indicates Robo2-labelled axons. Scale bar in (D1) = 200 μm for (B-D1) and 500 μm for (D2); scale bar in (G) = 200 μm for (E-G). Click here for file

Additional file 6 **Figure S5: TAG-1 positive commissural axons turn longitudinally instead of crossing the midline in Rig-1 mutant.** Midline crossing failure of TAG-1 positive axons in whole mount preparation of the hindbrain. (A,B) Ventral views of TAG-1 immunostained hindbrain from E11 wild-type (A) and *Rig-1 *homozygous mouse (B). Note many TAG-1 immunopositive axons near the ventral surface grow longitudinally on the ipsilateral side without crossing the FP (arrows). The scale bar in (B) is 100 μm and applies to (A,B). Rostral is to the top. Click here for file
