# Supplementary material for: Crucial roles of Robo proteins in midline crossing of cerebellofugal axons and lack of their up-regulation after midline crossing
Source: Neural Dev. 2008 Nov 5;3:29. doi: 10.1186/1749-8104-3-29 (PMC2613388; doi:10.1186/1749-8104-3-29)
Supplement: Additional file 1 — Supplemental information. Explanation of the procedures and specificity of the generated antibodies. [file 1749-8104-3-29-S1.pdf]

Supplemental Information

Since the antigens contain the Fc portion of IgG, we suspected that these antibodies might recognize the Fc portion only. To exclude this possibility, immunoblot analysis was performed with Robo-Fc proteins digested by Factor Xa, which cuts the protein into a Robo ectodomain and an Fc portion. Each Robo antibody recognized both the ~120kDa band of the Robo ectodomain and the ~40kDa band of the Fc region (data not shown). These results indicate that the antibodies raised against Robo1eFc, Robo2eFc and Rig-1eFc proteins indeed recognize the ectodomains of Robo1, Robo2 and Rig-1, respectively.

We first examined the specificity of these antibodies by immunohistochemical analysis using coronal sections of E14 rat hindbrain. While signals were detected in the rat medulla oblongata by the anti-Robo1 antibody (Fig. S1B), they disappeared when the antibody was pre-absorbed with Robo1eFc protein (Fig. S1B′). These signals, however, did not disappear when the antibody was pre-absorbed with Robo2eFc, Rig-1eFc, or Fc protein (data not shown). Likewise, signals detected by anti-Robo2 (Fig. S1C) or anti-Rig-1 (Fig. S1D) antibody disappeared only when pre-incubated with Robo2eFc (Fig. S1C′) and Rig-1eFc (Fig. S1B′) proteins, respectively. These results indicate that Robo1, Robo2 and Rig-1 antibodies specifically recognize Robo1, Robo2 and Rig-1, respectively.

The specificity of the antibodies was further confirmed by application of the antibodies to *Rig-1* knockout mice and *Robo1/2* double knockout mice.

Application of a Rig-1 antibody to hindbrain sections of *Rig-1* knockout mice caused no staining (data not shown). Although a Robo2 antibody caused no staining of *Robo1/2* double knockout mice preparation, Robo1 antibody caused some immunoreactivity (data not shown). This can be explained by a small amount of splicing over the gene trap insertion, which causes the presence of a small amount of wild–type Robo1 mRNA in *Robo1* knockouts (Long et al., 2004).
