# Supplementary figures and images for: Crucial roles of Robo proteins in midline crossing of cerebellofugal axons and lack of their up-regulation after midline crossing
Source: Neural Dev. 2008 Nov 5;3:29. doi: 10.1186/1749-8104-3-29 (PMC2613388; doi:10.1186/1749-8104-3-29)

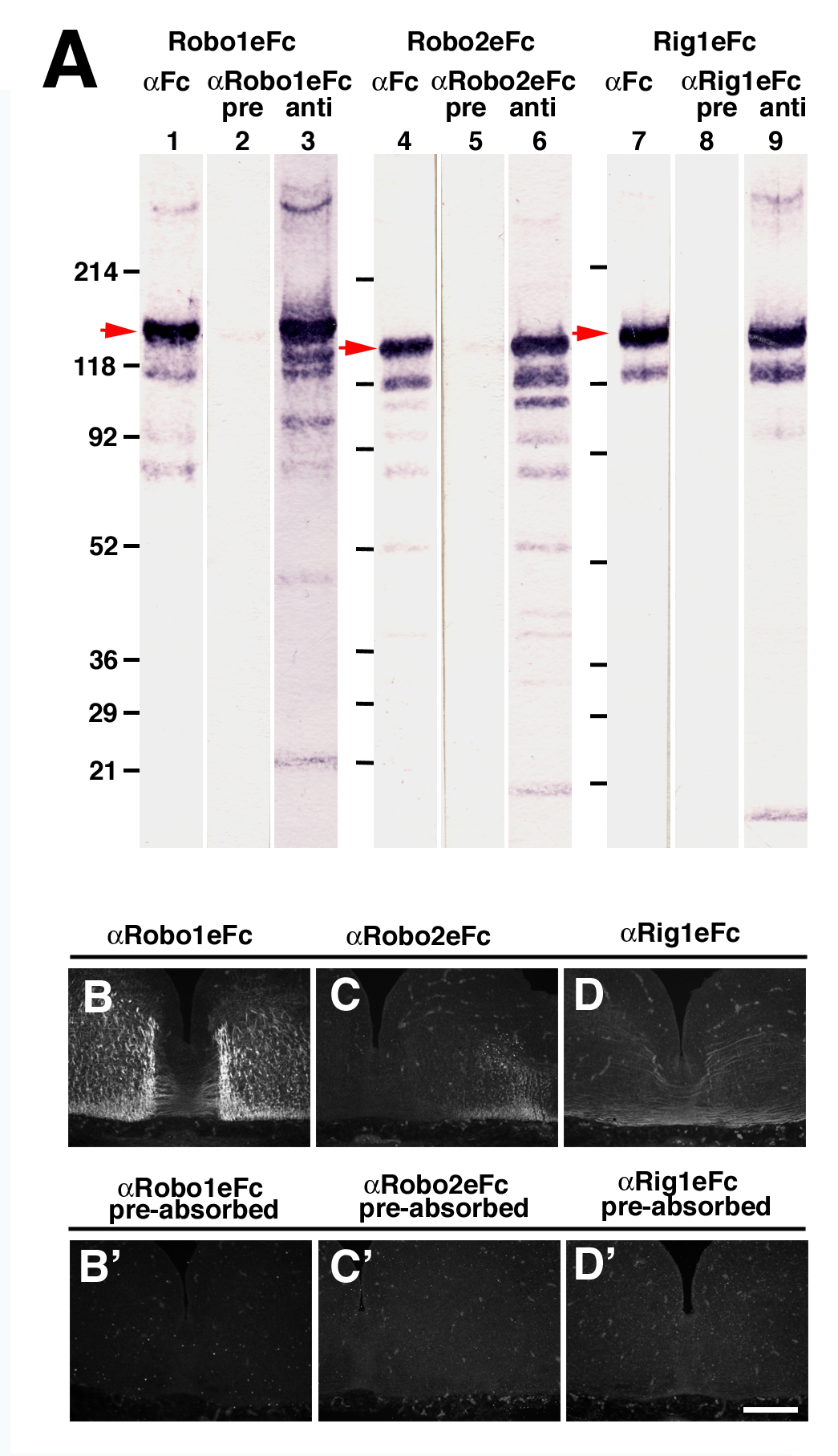

Supplement: Additional file 2 — Figure S1: procedures and specificity of the generated antibodies. Specificity of rabbit anti-rat Robo1 and Robo2 antibodies and anti-mouse Rig-1 antibody. (A) Robo1eFc (lanes 1–3), Robo2eFc (lanes 4–6) and Rig-1eFc (lanes 7–9) proteins were separated by SDS-PAGE and blotted onto a nitrocellulose membrane. To identify Robo proteins, the membrane was reacted with an antibody against the human IgG1 Fc region (lanes 1, 4 and 7), IgG fractions purified from pre-immune sera (lanes 2, 5 and 8) or antisera (lanes 3, 6 and 9). Arrows indicate the molecular weight of the corresponding Robo-Fc fusion proteins. (B-D) Cy3 immunofluorescence of coronal sections from E14 rat hindbrain stained by antibodies raised against Robo1eFc, Robo2eFc and Rig-1eFc. (B'-D') Same as (B-D) but the antibodies were pre-adsorbed by excessive antigens. Scale bar = 200 μm. [file 1749-8104-3-29-S2.tiff]

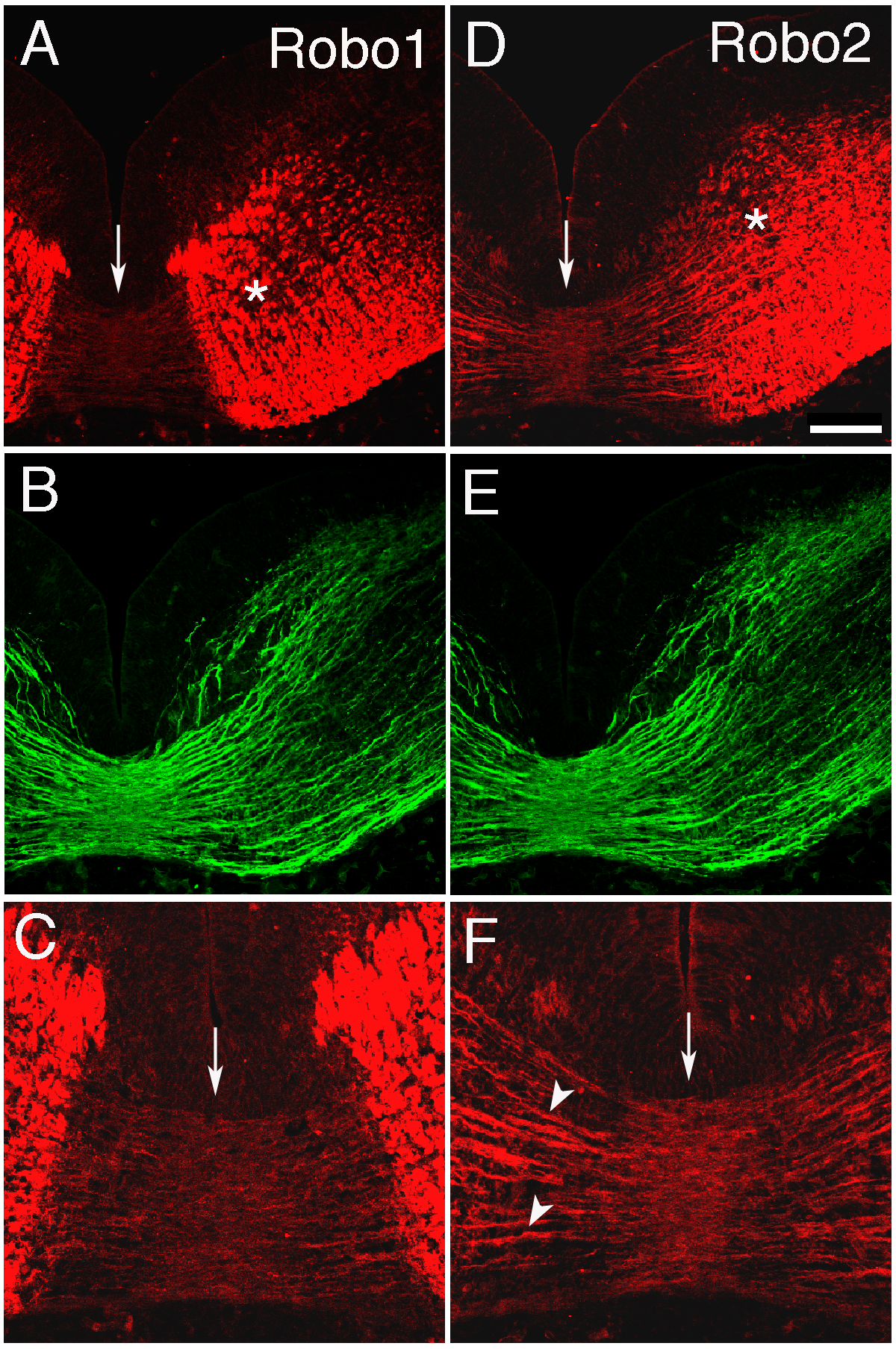

Supplement: Additional file 3 — Figure S2: immunostaining of coronal sections with Robo1, Robo2 and TAG-1. Robo1 and Robo2 immunoreactivies for circumferential axons in the midline region. (A-F) Immunostains for Robo1 (A,C), for Robo2 (D,F) and TAG-1 (B,E). Arrows indicate circumferentially growing axons in the midline. Asterisks show cross sections of longitudinally growing axons. Robo2 immunoreactive circumferential axons can be observed several distances from the ventral midline. These are unlikely to be post-crossing CF axons because CF axons make longitudinal turns in a region closer to the midline (Figure 7). Comparisons with immunostaining for TAG-1, which is expressed in hindbrain and spinal cord commissural axons before midline crossing [21], support the notion that CF axons express Robo1 as well as Robo2 (Figure 2B,E). Coronal sections of an E14 rat embryo. Scale bar = 150 μm in (A,B,D,E) and 75 μm in (C,F). [file 1749-8104-3-29-S3.tiff]

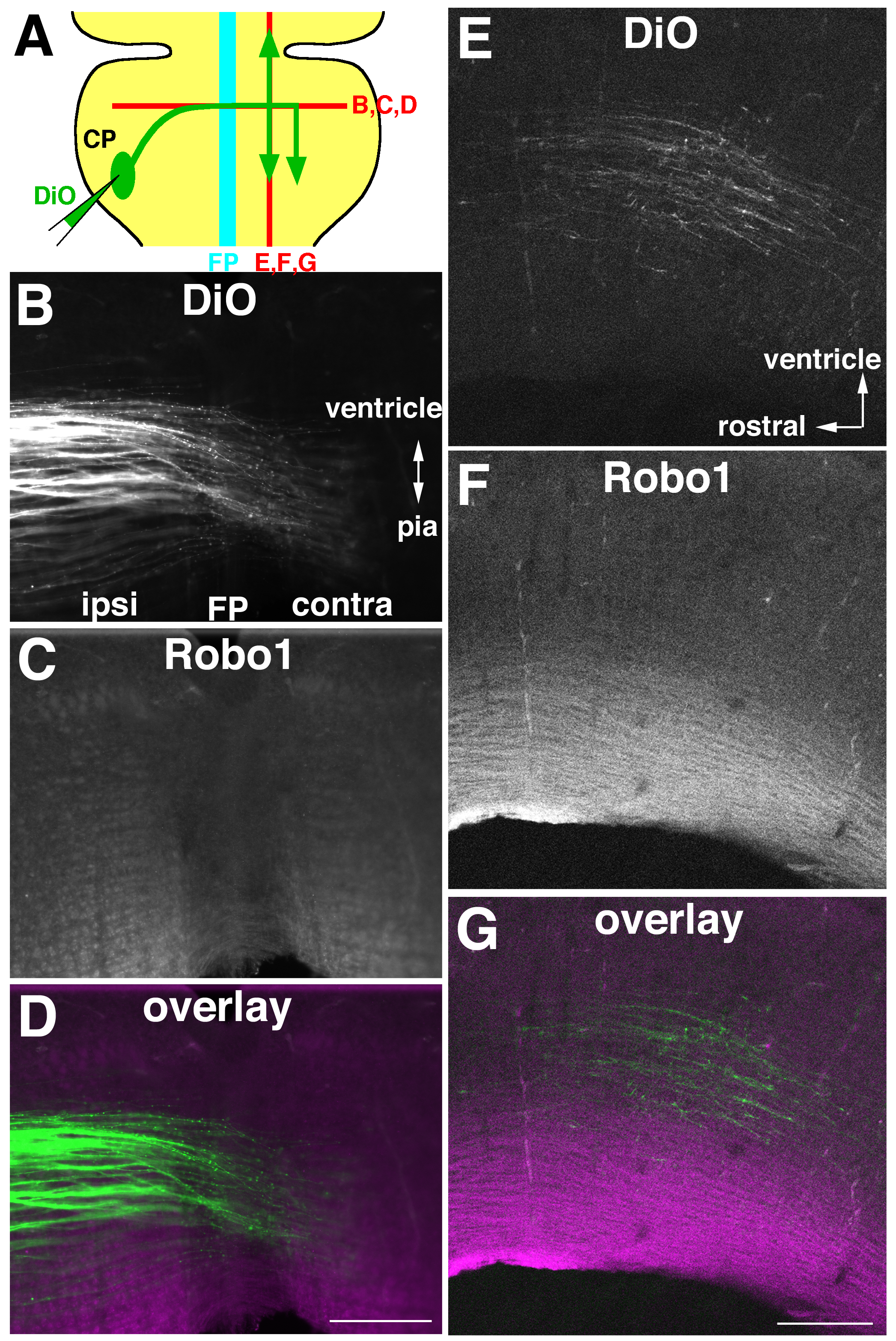

Supplement: Additional file 4 — Figure S3: segregation of Robo1 immunoreactivity and CF axon trajectory. Comparison of CF axon trajectories with Robo1 immunoreactive axons. DiO was injected into the CP of E16 flat, whole-mounted hindbrain after fixation. After allowing for DiO diffusion, coronal sections (B-D) or parasagittal sections (E-G) of the brain were made and immunostained for Robo1. (A) Schematic showing the trajectory of DiO positive CF axons (green). Red lines indicate planes of the section that correspond to designated panels. (B-D) DiO-labelled axons and Robo1 immunoreactivity in a coronal section. Ipsi, ipsilateral; Contra, contralateral. (E-G) Robo1 immunoreactivity and ascending DiO-labelled axons in the parasagittal section. In both planes, the Robo1 immunoreactive region was located more superficially to the region where DiO-labelled axons were found. Scale bar = 200 μm. [file 1749-8104-3-29-S4.tiff]

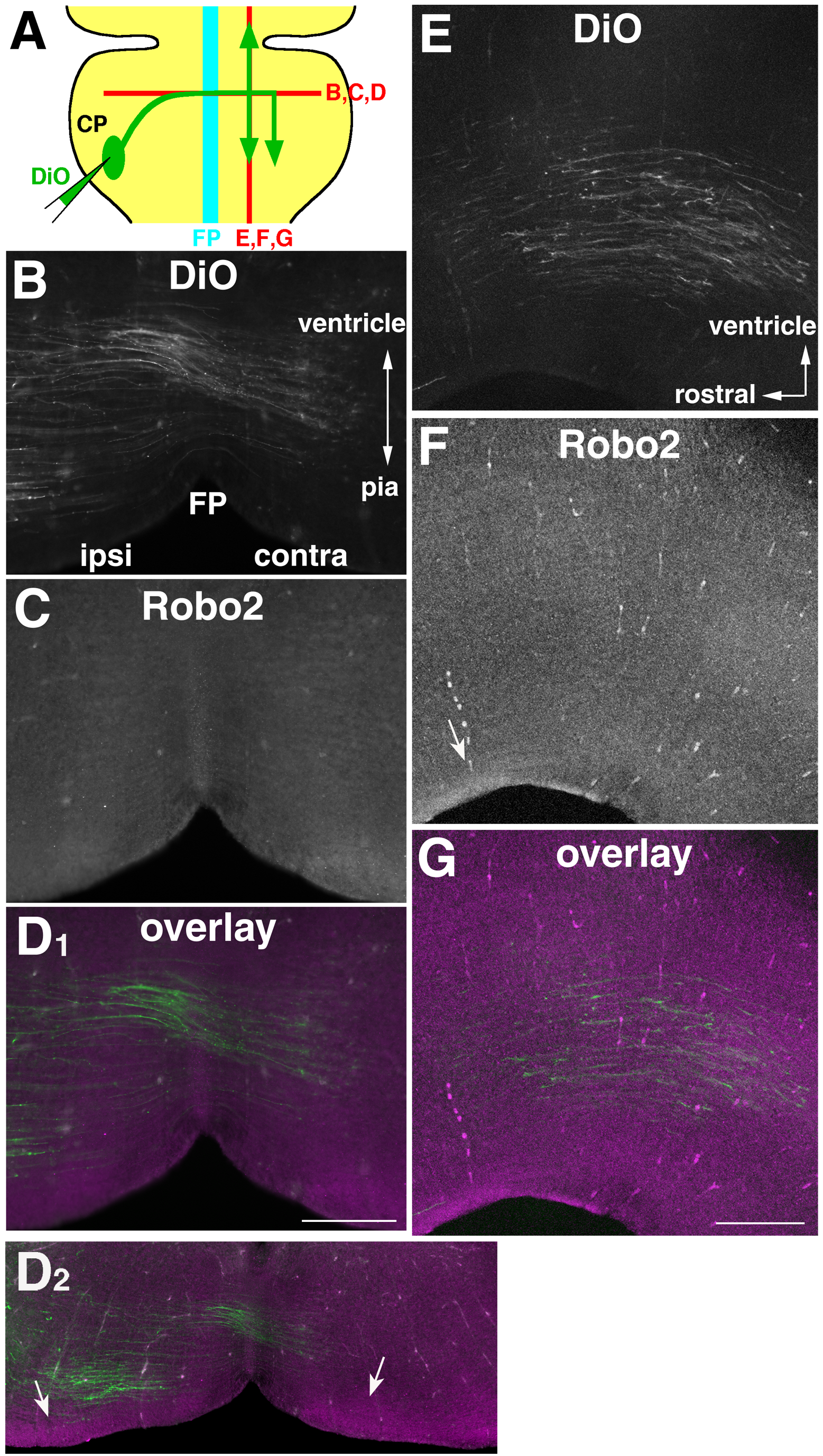

Supplement: Additional file 5 — Figure S4: segregation of Robo2 immunoreactivity and CF axon trajectory. Comparison of CF axon trajectories with Robo2 immunoreactive axons. E16 preparations were treated similarly to those in Figure S3 (Additional file 4) but immunostained for Robo2. (A) Schematic showing the trajectory of DiO positive fibres (green) and planes of the section (red). (B-D) DiO-labelled axons and Robo2 immunoreactivity in a coronal section. (D2) is a lower magnification view of (D1) showing Robo2 immunoreactive fibres running near the ventral surface (arrows). Ipsi, ipsilateral; Contra, contralateral. (E-G) DiO-labelled axons and Robo2 immunoreactivity in a parasagittal section. In both planes, Robo2 immunoreactivity, although weak, is located superficially to the region where DiO-labelled axons are found. White arrow in (F) indicates Robo2-labelled axons. Scale bar in (D1) = 200 μm for (B-D1) and 500 μm for (D2); scale bar in (G) = 200 μm for (E-G). [file 1749-8104-3-29-S5.tiff]

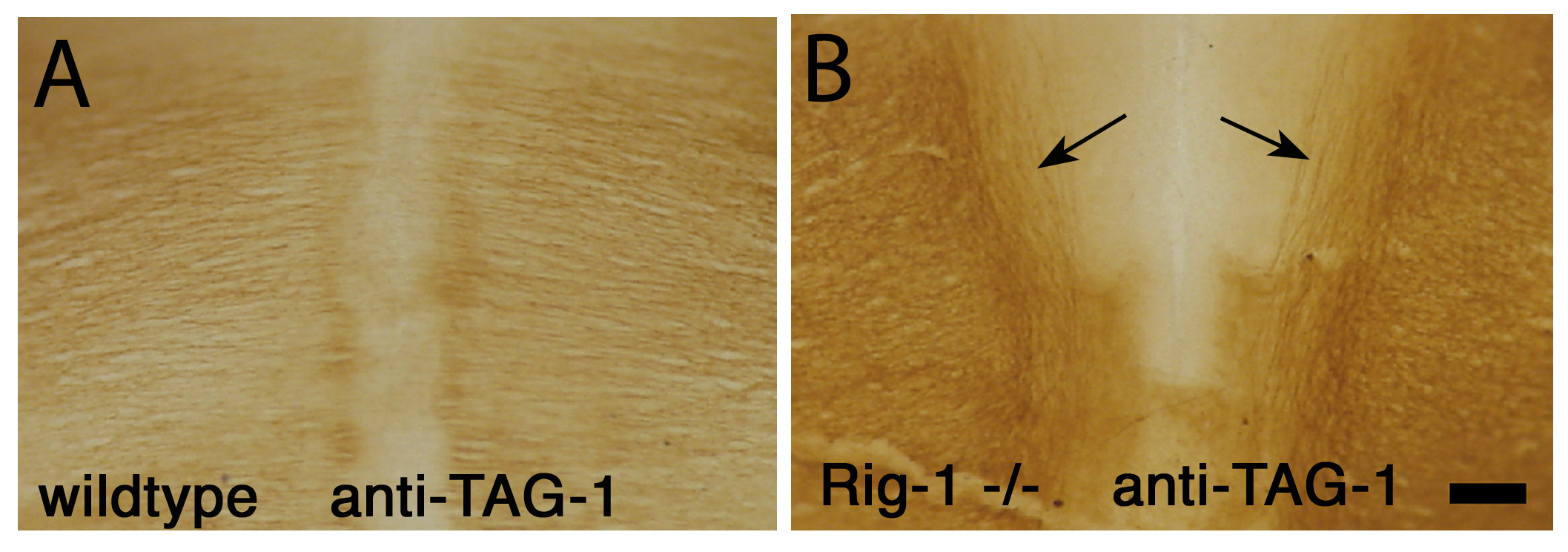

Supplement: Additional file 6 — Figure S5: TAG-1 positive commissural axons turn longitudinally instead of crossing the midline in Rig-1 mutant. Midline crossing failure of TAG-1 positive axons in whole mount preparation of the hindbrain. (A,B) Ventral views of TAG-1 immunostained hindbrain from E11 wild-type (A) and Rig-1 homozygous mouse (B). Note many TAG-1 immunopositive axons near the ventral surface grow longitudinally on the ipsilateral side without crossing the FP (arrows). The scale bar in (B) is 100 μm and applies to (A,B). Rostral is to the top. [file 1749-8104-3-29-S6.tiff]
